# Teaching to transform surgical culture: an educational programme and thematic analysis in a general surgery department

**DOI:** 10.1186/s12909-022-03941-3

**Published:** 2023-01-23

**Authors:** Madhav Sanatkumar Dave, Shahd Mobarak, Munir Tarazi, Christian Macutkiewicz

**Affiliations:** 1 Division of General Surgery, Manchester University NHS Foundation Trust, Oxford Road, Manchester, M13 9WL UK; 2 Department of Surgery and Cancer, Imperial College London, London, UK

**Keywords:** Medical education, Postgraduate education, General surgery, Hidden curriculum, Multidisciplinary, Thematic analysis

## Abstract

**Introduction:**

General surgery departments are busy, meaning educational opportunities may be sporadic. Clinical priorities can sometimes supersede teaching and trainees may feel alienated at the periphery of the working community. In this study, we demonstrate how a reflective, multidisciplinary general surgery teaching programme was established and use this to assess the impact of structured teaching on surgical doctors of all grades in the department.

**Methods:**

Twelve semi-structured telephone interviews were conducted with participants of varying grades. Transcripts were analysed using a grounded theory thematic analysis, revealing four themes: the value of teaching; learning as a community; barriers to successful training; and culture of surgery.

**Discussion:**

Teaching helped juniors construct healthy narratives around general surgery and encouraged a process of professional identity formation. Pairing junior and senior colleagues allowed both to develop their skills, and reflective learning revealed new learning opportunities. Transparency across the ‘community of practice’ was achieved and the programme helped juniors overcome negative stereotypes of intimidation embedded in the hidden surgical curriculum.

**Conclusion:**

Reflective, multidisciplinary learning can challenge the hidden curriculum and encourage team cohesion. A commitment to critical reflective teaching will be vital in cultivating surgeons of the future.

**Supplementary Information:**

The online version contains supplementary material available at 10.1186/s12909-022-03941-3.

## Introduction

### Context

Training the next generation of general surgeons is becoming an increasingly important endeavour. Discounting figures for breast, endocrine, transplant, miscellaneous or shared admissions with allied medical specialties, hospital episode statistics (HES) data from 2018–2019 reported just over 1.2 million hospital admissions to emergency general surgery teams in England alone [1]. Considering these are incomplete data, it is likely that these numbers greatly underestimate the true burden on services.

The organisational, operational and epidemiological challenges facing the UK’s National Health Service (NHS) mean that surgical practice, and hence training, are having to change quickly [2]. Furthermore, the ongoing COVID-19 pandemic has taken away many educational opportunities from trainees [3]. Training is often variable, unstructured, informal, sporadic, or even non-existent [4].

There are cultural problems too. Surgical trainees consistently report the lowest satisfaction among trainees and regularly work beyond contracted hours or expected levels of competence [5, 6]. High attrition rates are often secondary to significant workloads; a lack of training opportunities; a culture of hierarchy; a lack of emotional support in a learning community and a fragile professional identity without a sense of belonging [7]. Regular, interdisciplinary educational programmes have previously been suggested to support trainees in their development as general surgeons [8]. Such teaching programmes, it is hoped, can challenge the ‘hidden curriculum’.

### Theoretical underpinnings

This study is underpinned by a constructivist approach, meaning knowledge is considered an emergent, situated phenomenon [9, 10]. Such knowledge is communally held and enacted within a ‘community of practice’ [11]. Workplace learning is impossible without participation and social interaction, where novices move from the periphery of the working community to the centre as they gain experience [12, 13]. This is a process of socialisation and identity formation [14, 15]. Language, symbols and beliefs all mould the identity of the apprentice into that of an expert consultant surgeon [16].

The development of professional identity is affected by subconscious learning and tacit rules emerging from the ‘hidden curriculum’: the ‘unwritten rules’ [17–19]. The professional sphere can thus seem impenetrable to a novice. Critical reflection, both spontaneous and formal, can help trainees acknowledge the cultural norms that delineate the culture of surgery [20, 21].

This study aims to evaluate a reflective multidisciplinary teaching programme implemented in a general surgery department. We use participant extracts to shed light on the value of the programme, the benefits and barriers perceived by both teachers and learners, as well as the process of cultivating a new learning community.

## Methods

### Design and implementation of teaching programme

No structured teaching programme existed in the department, and learning was therefore ad hoc on ward rounds, in clinics or in theatre. A team was therefore formed comprising two junior doctors (MSD, SM), a surgical registrar (MT) and a consultant surgeon (CM). A needs assessment was undertaken by the team to develop learning objectives and a curriculum, using a combination of undergraduate and postgraduate curricular learning outcomes, published standards and personal experience [22]. Objectives of the programme included establishing weekly peer-led teaching; the development of a multi-disciplinary learning community; and the cultivation of a culture of reflection through critique of surgical practice and literature.

Junior speakers were paired with a registrar or consultant and were asked to develop teaching content under their supervision. All content was approved by CM prior to delivery. Topics were taken from the fields of upper gastrointestinal, colorectal, hepatobiliary, emergency general, vascular, urology and transplant surgery. Sessions were held for 45 min every Wednesday afternoon during lunchtime. Resources and presentations were pooled into an openly accessible drive and learning was both summarised and promoted via Twitter [23].

Emphasis was placed on encouraging critical reflection on clinical practice to reveal beliefs, preferences and assumptions. This also helped to establish existing knowledge and therefore create dissonance, leading to further learning [24]. After each session, all attendees were asked to complete a written feedback form, and tutors debriefed on their session with the team. This helped them to cultivate the metacognitive aspects of reflection which improve the educator’s own teaching ability.

Speakers and attendees included medical students, surgeons, physicians, radiologists, intensivists, clinical coders, specialist nurses, dieticians, endoscopy nurses and pharmacists. Video- and multimedia assisted learning methods were employed, as well as two practical skills workshops teaching basic suturing and knot-tying.

### Data collection

Participants in this study were identified using purposive sampling and included individuals who had taught or attended at least three teaching sessions.

Subjects were emailed a brief description of the study. Both verbal and written consent were gained prior to interviewing. The interview schedule was developed and agreed upon by MSD and SM (see Additional file 1: Appendix 1). Questions were piloted through three initial interviews with a consultant and two juniors. These were excluded from the analysis. This revealed the importance of asking open-ended questions on the individual’s involvement before moving onto questions about the teaching programme in the context of the department and learning opportunities. Pilot interviews revealed that these questions were best asked after rapport had been established. Questions were then rephrased, refined and standardised by MSD, allowing the identification of ‘key questions’ [25, 26].

Semi-structured interviews were conducted over the phone at a mutually agreeable time and audio-recorded by MSD [27]. Interviews were conducted in a private space where participants were free from disturbance or clinical commitments, either at home or in their office. While nominal groups had been the chosen method of data collection, the study was affected by the COVID-19 pandemic which precluded face-to-face data collection. At the time of interviewing, the teaching programme had been running for 14 months.

Twelve interviews were conducted in total, ranging from 10 to 34 min in length. Excluding the shortest interview (an outlier), median interview time was 23 min. Saturation was defined as the emergence of no new concepts and was reached by the ninth interview, precluding the need for further theoretical sampling [28]. Participants included medical students, radiologists and even dieticians. Participant characteristics can be found in Additional file 1: Appendix 2.

Audio-recordings were undertaken on a password-protected mobile phone device, before being anonymised using a numerical code and transcribed. Since interviews were conducted by MSD, only MSD had access to the codes revealing participant identities. All files were password-protected and stored on an encrypted NHS-server.

### Data analysis

Data were analysed using an inductive grounded-theory approach [29]. Grounded-theory allows for deep engagement with the data, flexibility, and the ability to compare voices within the data to generate theory. This was well-suited because the study aimed to analyse how participants were affected by the teaching programme, and how they constructed their experiences of it as a member of a wider surgical team.

Audio-recordings were anonymised and transcribed verbatim by both MSD and SM. Each interview was assigned a numerical code according to the order in which they were conducted.

Transcripts were read in accordance with audio-tapes before being member-checked. They were then read broadly to identify categories emerging *prima facie*, and assigned memos helped direct the formation of codes and categories. A more detailed reading allowed for more elaborate memos to be attached. This method helped define saturation, meaning further interviews were not required [30].

A total of 28 codes were developed by MSD and SM. Emphasis was placed on language, comparisons, assumptions, metaphors, repetitions, and *gerunds *[29]. Codes were read and checked in accordance with the transcripts by MT and finalised into four themes by MSD. These were further checked for accuracy and re-read in the context of the transcripts by both SM and MT. Further theoretical sampling was not deemed necessary to further develop the themes.

## Results

Four themes were developed and are explored with data extracts below. Interview number is indicated numerically in brackets.

### The value of teaching

The teaching programme was considered a valuable addition to the department.

“Before this was set up, there was no departmental teaching” (12)

“I found these sessions to be quite useful because on my general surgery job there wasn’t always a lot of teaching other than sporadic here and there on the ward round” (6)

The teaching programme challenged clinicians, encouraging them to develop as educators. Teaching helped speakers deepen their own knowledge on the subject.

*“In order to be able to go and teach a subject you need to have the abilities to synthesise lots of information and deliver that information in an effective way, which in turn will also help [your] clinical practice.” (3)*

Sessions provided context to daily practices.

*“It has added interest and understanding in what people were doing rather than just doing jobs without understanding why they were doing jobs. They were understanding the principles behind, [everything from the] pathology to pathophysiology to clinical management…the bigger picture.” (4)*

In particular, the reflective approach to learning helped cultivate a sense of agency. Junior doctors began portraying themselves as both self-directed learners and self-efficacious in bringing about change.

*“When people are moving from a university-based education to work, they [junior doctors] expect everything to be delivered to them on a plate, whereas when you go to work, you start having to be the producers of content, rather than just constantly receiving…they start realising that they can impact organisations themselves.” (3)*

The programme also provided opportunity for attendees. Junior doctors saw it as a place to access specialist knowledge from experienced surgeons and to further their career aspirations.

“You don’t really get any teaching…and I don’t know where those resources are that would explain it quite so simply and quite so accessibly [as the teaching programme]” (5)

Senior clinicians on the other hand saw it as an opportunity to gain experience in leadership and mentoring.

“More senior trainees can hone their teaching skills and apply things we may have learnt in postgraduate medical certificate diplomas.” (9)

The programme encouraged juniors to acknowledge their agency in learning and provided opportunity for development, irrespective of grade.

### Learning as a community

A flattened hierarchy in a learning community does not always come about organically. Participants stressed the importance of cultivating this and often spoke of its benefits.

“Being in a room with all different levels of doctors…we don’t really get that at all.” (1)

Reflective interaction between juniors and seniors allowed for richer learning across grades and specialties.

*“…[teaching was an] especially good forum for conversation and questioning…the senior trainees and consultants joined in to make it a much more valuable educational experience than just a standalone lecture” (5)*

The involvement of doctors from all grades meant that fresh light could be shed on existing knowledge. Each practitioner could bring their previous experiences and hence their individual perspective to the discussion.

“I quite like it where it is varied, where one week it’s a consultant the next week it’s an F1…they teach differently, and they teach different kinds of things” (10)

Most participants favoured a mixed teaching approach in which sessions were primarily led by juniors and supported by consultants. Appropriately challenging the trainee empowered them to take on responsibility and improved the quality of the content. Supportive consultants were able to facilitate this while identifying and rectifying any errors.

“The content was at a higher level which was actually why it was good teaching…I feel like it should be above our grade to help push us forwards.” (5)

Furthermore, the involvement of a senior clinician can guide the identity formation of a junior doctor into a surgeon, shaping their training and progression.

*“When you see your consultants in those teaching sessions giving feedback or giving pointers, it empowers you to feel like they actually care. It is easy to feel like they don’t care about your career or about your training as a doctor. But when you’ve got a formal plan of teaching that happens every week, you see…that they [consultants] do care about your career progression and your training.” (9)*

A safe, supportive learning environment can also demystify those at the centre of the community of practice and help foster transparency across the team.

*“I even remember meeting consultants who I never would have even spoken to otherwise but because we were put in that environment, I did get chatting to them. And [they] had some really amazing bits of wisdom” (1)*

*“You have F1s and consultants all sitting down together, all having lunch together, all chatting together. [This] slightly breaks down that hierarchy and the interaction between the F1 and the consultant or SHO and consultant isn’t as serious” (12)*

Cultivating a community centred on reflection meant that reciprocal learning could take place. Registrars acknowledged that they too had learnt something from the junior foundation doctors.

*“I speak for a few other registrar colleagues when we say, a few of us learnt a lot of up-to-date information that we wouldn’t have known before we started that teaching session. And that was delivered by an F1 [junior] doctor.” (9)*

Similarly, other members of the multidisciplinary team were able to demonstrate how their contributions holistically improved patient care.

*“The roles of the other MDT [multidisciplinary team] members aren’t always fully appreciated…I think often there’s a lack of understanding from doctors. I think having that opportunity to be able to explain your role in a bit more depth and detail…improves outcomes.” (7)*

### Barriers to successful training

Study subjects identified several barriers which can preclude effective learning. These were often described in general terms rather than applied specifically to the teaching programme.

The most cited barrier was a lack of time. Junior doctors were often portrayed as being too busy to be able to attend and organise a teaching programme.

“If you’re a junior doctor, you’ll be running around like a madman, you’re always hungry and needing the toilet!” (1)

“Finding a time when everyone’s going to be free. Everyone’s busy. Everyone’s got different schedules, so finding that time is going to be difficult” (11)

Registrars and consultants on the other hand were criticised for not investing their time or appropriately prioritising educational activities.

“Consultants don’t get paid for it [teaching]. Unfortunately for some, that’s what they’re all about. So, if they don’t have the time in their job plans to teach, then they won’t bother.” (2)

“But the truth is [consultants] overall are not interested. This is the truth.” (4)

Lastly, inadequate facilities can affect the continuity or quality of teaching.

“You know there might be some sort of bureaucracy in the way” (3)

“The venue at that particular time wasn’t great because it was quite a small room so there were a lot of people sort of standing up and listening to what was going on” (8)

Time constraints, clinical workload, inadequate facilities and a lack of consultant interest can all negatively affect teaching practices in a department. This can have a significant impact on the way junior doctors perceive general surgery and develop their professional identity.

### Culture of surgery

A significant number of participants spoke of a wider culture of surgery in which the teaching programme was situated. While this was rarely in relation to our programme directly, this theme defined the unspoken barriers that prevent peripheral participants from engaging with surgical education.

General surgeons were often portrayed as stereotypically intimidating.

“There’s always that stereotype that they’re very nasty, cutthroat people” (12)

“There’s this big attitude of ‘oooh they’re surgeons, they’re scary’…I do know why it continues.” (1)

The latter subtly illustrates that the persistence of these stereotypes depends at least partially on their reflection of the truth. This was acknowledged with much hesitation. In many instances, previous negative experiences of surgeons shaped a wider perception of surgery.

“There’s that one experience – because it’s such a shocking experience – that story just gets retold and retold and retold. So the…fear of it passes on. It gets continued, it gets propagated.” (1)

Similarly, a culture of teaching and learning through fear was described alongside these stereotypes. This can be particularly harmful as it undermines trainee confidence.

*“I think general surgeons in my experience tend to be quite patronising in their teaching methods…they can also be quite mean in their teaching methods…[this can] make you hate surgery even more than you already do.” (10)*

“Learning by humiliation…I acknowledged that I had that sort of teaching, but I also realised that it is not the best way to get things out of people.” (2)

While the former quote demonstrates how these experiences can shape lasting perceptions of the specialty, the latter shows how surgeons are starting to acknowledge the damage these interactions can inflict.

*“I feel that the F1s and F2s [junior doctors] if they come and say that I want to be a surgeon, they will ‘quote unquote’ “teach them”. If they come and say I want to be anything else, it’s almost seen as a bit of a joke…And they always pre-decide who’s going to be a surgeon, who’s not going to be a surgeon, who they can be bothered to teach and who they can’t be bothered to teach.” (8)*

These insidious, unwritten cultural rules can be particularly difficult to challenge and change.

*“Most people just get on with things, they just swallow their pride and get on with things. Because there’s almost an expectation that that’s what you’re supposed to do…But actually, the more that we don’t do that [challenge behaviour], the more that we perpetuate that sort of negative behaviour. But I think that’s a really difficult thing to try to fix and it probably requires massive cultural change amongst the general medical and surgical community” (9)*

Lastly, the teaching programme provided an opportunity to reassure juniors that an environment of humiliation was neither necessary nor appropriate. Participants acknowledged that a culture of fear no longer had a place in modern surgical practice. They proposed reflective, multidisciplinary surgical education as a driver of change.

*“I think it’s the whole aspect of junior-led teaching also helps break down those stereotypes that have formed over the many many decades…And if they [consultants] see that there are juniors that are keen to learn more about surgery and even teach then I feel that that would be a mutually beneficial relationship between the seniors and the juniors” (3)*

Critically reflective, inclusive methods of teaching could help deconstruct hierarchy and lead to lasting change.

## Discussion

Participants of the study acknowledged various benefits to the teaching programme. Extracts show that teaching helped junior doctors contextualise their daily clinical practice. By identifying learning needs and creating dissonance, trainees developed a sense of agency and self-efficacy [24, 31]. Furthermore, interdisciplinary learning from participants of various backgrounds and grades contributed a variety of perspectives, which demonstrably deepened learning.

We maintained a high standard of teaching by pairing junior and senior clinicians together. This propelled junior doctors beyond their usual capabilities [32]. The interactions bestowed opportunities which would not otherwise present themselves, such as access to the expertise of consultants, career advice and mentoring experience. Reciprocal learning compelled more senior trainees to acknowledge that all members of the department had valuable contributions to make, stimulating debate and team cohesion, all while breaking down hierarchy. This could improve the efficiency of the team, the satisfaction of its members and ultimately the quality of the care provided.

Developing a reflective community of practice demystifies its practices and its senior members. Individuals situated in their workplace culture can be blind to its unwritten rules or rituals [33]. Communal reflection on evidence and departmental practice can provide valuable insights, helping trainees recognise the messy nature of situated surgical practice [20, 21, 34]. In the same way, those at the periphery of the community may not feel sufficient proximity with central figures to identify with them. Those at the centre of the community of practice may fail to acknowledge the barriers that stand in the way of the juniors and their learning. By collaborating and sharing reciprocal perspectives, we showed that greater proximity and transparency can be achieved throughout the team.

Supportive consultants and safe learning environments are crucial in the construction of narratives and professional identities. The formation of professional identity is a complex process, dependent upon relationships, experiences and emotions [15, 35, 36]. Our study shows how an early experience of humiliation can shape future perceptions of the profession. Stories and stereotypes often act as powerful means of perpetuating the hidden curriculum [37]. Without safe learning environments, junior doctors are unlikely to construct healthy narratives surrounding general surgery and may not identify with surgery at all [14]. That said, our data also shows how teaching can be a powerful tool with which departmental discourse can be rewritten. Challenging formed conceptions with reflections and a non-hierarchical teaching programme can help transcend established narratives on general surgery [38].

Barriers to the implementation of a teaching programme are varied and difficult to change. This study confirmed that a lack of time and increasing clinical demands placed on doctors can mean that doctors find it difficult to even attend, let alone organise departmental teaching themselves [39, 40]. Facilities may be lacking, and continuity can therefore be compromised [41]. While these barriers were specific to our efforts in our institution, it is likely that trainees elsewhere have similar concerns. Furthermore, consultant interest was at times reported as a scarce resource. Participants emphasised that unless a commitment to actively overcome barriers is made, educational activities are unlikely to be a workplace priority. As we have shown, this is detrimental to everyone. Unfortunately, logistical barriers are themselves inextricably tied to barriers in attitude and culture. Until these are changed, progress, if any, will be slow.

Our data demonstrates that power and hierarchy remain central to surgical education. Participants recognised that access to the community of practice depended on socialisation processes. Sometimes these involved intimidation and humiliation. Some participants drew on previous experiences, citing stories; many admitted that while most consultants saw their trainees as investments, an expectation to tolerate intimidating behaviour was at times the norm. This concerning culture can form a significant and impenetrable barrier to the cultivation of future surgeons. That said, our data shows that collaborative and reflective teaching could help transcend these narratives. Our programme helped deconstruct narratives of hierarchy; widened access to surgical knowledge; and inspired local change. Surgeons and surgical trainees have a responsibility to cultivate educational spaces in which power and hierarchy can be challenged and redefined.

Our study is not without its limitations. Data collection through telephone interviews meant that non-verbal cues were lost, and the interviewer depended upon verbal prompts to guide the participant. Virtual face-to-face data collection through an online platform such as Zoom may have limited the effects this had on data quality. Bias may have been introduced as the interviewer also transcribed and analysed the data, though member checking was used to alleviate the effects on the outcomes. Grounded-theory does not aim to produce generalisable theory and hence conclusions are intervention- and institution-specific. Conclusions could have been further strengthened by conducting an anonymous participant survey, further validating the results. Nevertheless, conclusions, though not necessarily generalisable, could help pave the way to larger multi-centre studies examining how surgical training can be improved on a bigger scale through teaching. Participants may have acted differently knowing that they are being interviewed. Feedback forms were intended to be used in the thematic analysis but were often poorly and hurriedly completed and did not provide any substantial data. Lastly, it is likely that the most important elements of the culture of surgery remained unspoken. Opinions too threatening or controversial to share will no doubt have been omitted from this analysis [33]. Participants will have been conscious of the position they occupy in the interview dynamic and while an informal interviewing approach was taken, there is no doubt that participants may not have vocalised their true opinions on some topics [27]. This may have been exacerbated by the fact that researchers were peers and not mere interviewers, meaning both may have shared blind spots given they occupy the same social space. However, such proximity between participant and researcher also helped develop rapport. A shared understanding was easier to cultivate and participants may have felt comfortable to express themselves freely without having to allude or suggest. This methodological flaw may therefore have been a strength in some interviews and a weakness in others.

Future directions for the teaching programme include further use of social media and immersive simulated learning to acclimatise juniors with the theatre environment. Though virtual learning was not employed in this study, an online journal club with a virtual discussion board may be of value alongside virtual teaching presentations. Live webinars may be logistically difficult to attend through the working day for juniors but allow a degree of flexibility for learners; conversely pre-recorded webinars may preclude live debate but nonetheless be an important addition to a bank of online, accessible general surgery resources.

Our teaching programme has illustrated that education remains a powerful catalyst for change. In a busy tertiary department with a significant workload, building educational regularity and commitment was challenging. In some ways, the theatre environment relies on hierarchy and a clear division between the expert and the novice [8]. That said, without learning relationships and transparency across the team, juniors at the periphery fail to relate to their senior peers and may subsequently fail to progress. Our data shows that negative connotations and a culture of intimidation can suffocate learning. Participants of all grades and specialties benefitted from the multidisciplinary and reflective approach. By encouraging learning relationships between junior and senior surgical doctors, reciprocal learning and team building can occur constructively. Furthermore, the difficult process of professional identity formation can be facilitated while challenging the hidden curriculum, in which juniors may feel so alienated that they are no longer are able to see themselves as surgeons. Our educational programme has brought lasting cultural change to the department and encouraged collaborative learning between professionals of all grades and specialties.

## Conclusions

General surgery departments are busy and clinical workload often takes precedence over surgical education. That said, our thematic analysis shows that reflective, multidisciplinary teaching is well received and that clinicians are aware of the benefits of a community of practice. Educational communities can help both individuals and departments rewrite the narratives that may be associated with general surgery.

## Supplementary Information

**Additional file 1.**

## Data Availability

The transcripted data used and analysed during the current study are available from the corresponding author on reasonable request. Audio files not available to protect confidentiality of study participants.
