# Supplementary material for: Teaching to transform surgical culture: an educational programme and thematic analysis in a general surgery department
Source: BMC Med Educ. 2023 Jan 23;23:51. doi: 10.1186/s12909-022-03941-3 (PMC9869620; doi:10.1186/s12909-022-03941-3)
Supplement: Supplementary file 1 — Additional file 1. [file 12909_2022_3941_MOESM1_ESM.docx]

Legend

[Appendix one (interview schedule) 2](#_Toc109318375)

[Appendix two (participant characteristics) 3](#_Toc109318376)

# Appendix one

- Tell me about your experience of the general surgery teaching programme
  - Your involvement
  - Good and why
  - Bad and why
  - Anything particularly memorable?
  - Would you change anything? If so, why?
    - Who should be teaching?
    - What should they be teaching?
    - Which format and for whom?
- Is having a structured teaching programme important?
  - Why wasn’t there one before? Do you think there are barriers?
  - How do we overcome these barriers?
  - Who should it be for and who should be teaching?

# Appendix two

Participant characteristics:

| **Grade** | **Number** |
| --- | --- |
| Medical student | 2 |
| Foundation doctor | 3 |
| Core surgical trainee | 1 |
| General surgical registrar | 2 |
| Consultant surgeon | 2 |
| Consultant radiologist | 1 |
| Dietician | 1 |

Male = 8 female = 4
